# String-pulling in the Goffin’s cockatoo (*Cacatua goffiniana*)

**DOI:** 10.3758/s13420-020-00454-1

**Published:** 2021-01-22

**Authors:** Birgit Wakonig, Alice M. I. Auersperg, Mark O’Hara

**Affiliations:** 1 University of Vienna, Althahnstraße 14, 1090 Vienna, Austria; 2 Messerli Research Institute, University of Veterinary Medicine Vienna, Veterinaerplatz 1, 1210 Vienna, Austria

**Keywords:** Physical cognition, Problem solving, Perception, Mental representation, Sensorimotor skills

## Abstract

Goffin’s cockatoos, a parrot species endemic to the Tanimbar Islands in Indonesia, demonstrate remarkable cognitive skills across various technical tasks. These neophilic extractive foragers explore objects with their beak and feet, and are skilled in several modes of tool use. In this study, we confronted the animals for the first time with a vertical string-pulling setup, including a set of classic and novel controls. Nine of the 12 subjects, two of which were subadults, immediately interacted with the single-string task, with seven individuals successfully obtaining the reward on their very first attempt. Four different double string discrimination tests with varying spatial relations were used to assess the Goffin’s cockatoos’ apprehension of basic physical task properties. We found significant differences in performance between the respective experimental conditions, as well as the development of side biases. The results suggest that while the birds seem to consider simple cause–effect relationships, there is no evidence for a mental representation of the causal mechanisms underlying the string-pulling tasks, as subjects failed the crossed strings condition out of immediate sight. Finally, we provide suggestions on improving the methodology, and discuss our findings in regard to the Goffin’s cockatoo’s ecology.

The string-pulling paradigm is an important benchmark test in comparative cognition that has been used to address various and often overlapping cognitive abilities, such as Gestalt perception, means–end understanding, causal reasoning, and insight (e.g., Köhler, 1925; Piaget, 1952; Taylor et al., 2010; Wang et al., 2019; Wasserman, Nagasaka, Castro, & Brzykcy, 2013). As the basic task is highly versatile, a multitude of string-pulling task versions are still widely applied to address various questions in multiple species, ranging from birds to mammals (including humans), and even invertebrates (Alem et al., 2016; Brown, 1990; Buttelmann, Carpenter, Call, & Tomasello, 2008; Chapman & Weiss, 2013; Jacobs & Osvath, 2015; Plotnik, Lair, Suphachoksahakun, & De Waal, 2011; Range, Möslinger, & Virányi, 2012; Riemer, Müller, Range, & Huber, 2014).

The basic setup of the string-pulling paradigm can easily be adjusted into different versions, depending on the target species and the purpose of the study. For example, the string orientation can be chosen to appear horizontal or vertical; when testing parrots, predominantly vertical string-pulling setups have been used (see Jacobs & Osvath, 2015), while horizontal arrangements have largely been employed with mammals, invertebrates and other bird species (e.g., Dücker & Rensch, 1977; Hofmann, Cheke, & Clayton, 2016; Obozova & Zorina, 2013; Taylor, Knaebe, & Gray, 2012’ but see Auersperg, Gajdon, & Huber, 2009; de Mendonça-Furtado & Ottoni, 2008; van Horik & Emery, 2016, for exceptions in testing parrot species). Other methodological variations include the alteration of string number and pattern. Tests with a single string are used to ascertain whether subjects possess the necessary sensorimotor skills to acquire the food reward at the end of the string (Altevogt, 1953). Multiple strings are used to investigate means–end understanding, for which four criteria have been proposed that must be met: (1) goal-directedness, (2) no proximity errors (choosing the string closest to the reward), (3) flexible solutions, and (4) no dependence on immediate feedback (Jacobs & Osvath, 2015). Using multiple strings can establish whether the pulling behavior is goal-directed or intrinsically rewarded (Hofmann et al., 2016), while introducing more complex string patterns, such as slanted, elongated, crossed, or double-crossed, can control for proximity errors, as well as allowing the investigation into the roles of visual and proprioceptive feedback (Hofmann et al., 2016; Schuck-Paim, Borsari, & Ottoni, 2009; Taylor et al., 2010).

A more recent addition to the classic string-pulling tests has been the introduction of visually restricted conditions (Gaycken, Picken, Pike, Burman, & Wilkinson, 2019; Molina, Cullell, & Mimó, 2019; Taylor et al., 2010). One way to restrict the view of the reward during pulling is to insert an occluder between the string’s anchor point and the reward (Molina et al., 2019; Taylor et al., 2010). In a study involving New Caledonian crows (*Corvus moneduloides*), one individual solved the task, while overall results were inconclusive after several trials (Taylor et al., 2010). Similarly, African grey parrots (*Psittacus erithacus*) did not solve the visually restricted conditions (Molina et al., 2019), and thus both studies indicate that subjects relied on proprioceptive feedback rather than more sophisticated cognitive processing. An alternative method is a task setup that requires subjects to pull a string in a downward facing direction in order to move the reward upward, by leading the strings over a pivot (Heinrich & Bugnyar, 2005). In order to limit visibility, a platform is installed for the subjects to stand on, preventing immediate visual access to the end of the string (Gaycken et al., 2019). After initial exposure to a standard string-pulling paradigm, in which the string was pulled simply upwards, several ravens (*Corvus corax*) successfully transferred their previous experience to the counterintuitive action of pulling downward. Therefore, the authors argued that some functional understanding, beyond mere operant conditioning, must have been employed in this task (Heinrich & Bugnyar, 2005). However, green-winged macaws (*Ara chloroptera*) failed to solve a similar transfer (Gaycken et al., 2019).

One of the first studies to systematically investigate string-pulling in parrots was an experiment conducted with four naïve African grey parrots by Irene Pepperberg (2004), to whom this special issue is dedicated (see Fischel, 1936; Funk, 2002; Rensch & Dücker, 1977, for other early studies). In her study, Pepperberg (2004) found that while language-naïve individuals would spontaneously perform the steps necessary for coordinated pulling, two language-trained grey parrots vocally requested the reward from the human experimenter, which was suggested as possibly representing a form of understanding of certain task properties. This study focused on the parrots’ spontaneity in pulling a single vertical string provided from a T stand and proposed that the language-trained subjects tried to engage in communication as a problem-solving strategy, thus exhibiting a transfer of behavioral patterns from one situation to another, which is considered a hallmark of intelligent behavior. Whereas the parrots who were not language trained successfully performed the string-pulling action sequence of pulling, stepping, and repeating, so as to obtain the reward at the other end of the single string and thus expressed spontaneity in the single string-pulling task.

Various psittacids have since been studied in different string-pulling studies (Table 1; see Jacobs & Osvath, 2015). While most parrots seem to be able to solve the basic string-pulling task, the crossed-string condition in particular seems to pose great difficulties for the majority of parrots. Only kea (*Nestor notabilis*), spectacled parrotlets (*Forpus conspicillatus*), galahs (*Eolophus roseicapilla*), and African grey parrots seem to be able to, at least partially, solve this condition (Krasheninnikova, 2013; Krasheninnikova, Bräger, & Wanker, 2013; Molina et al., 2019; Werdenich & Huber, 2006). However, the view of the strings was only fully restricted for the African grey parrots (Molina et al., 2019).

**Table 1:**
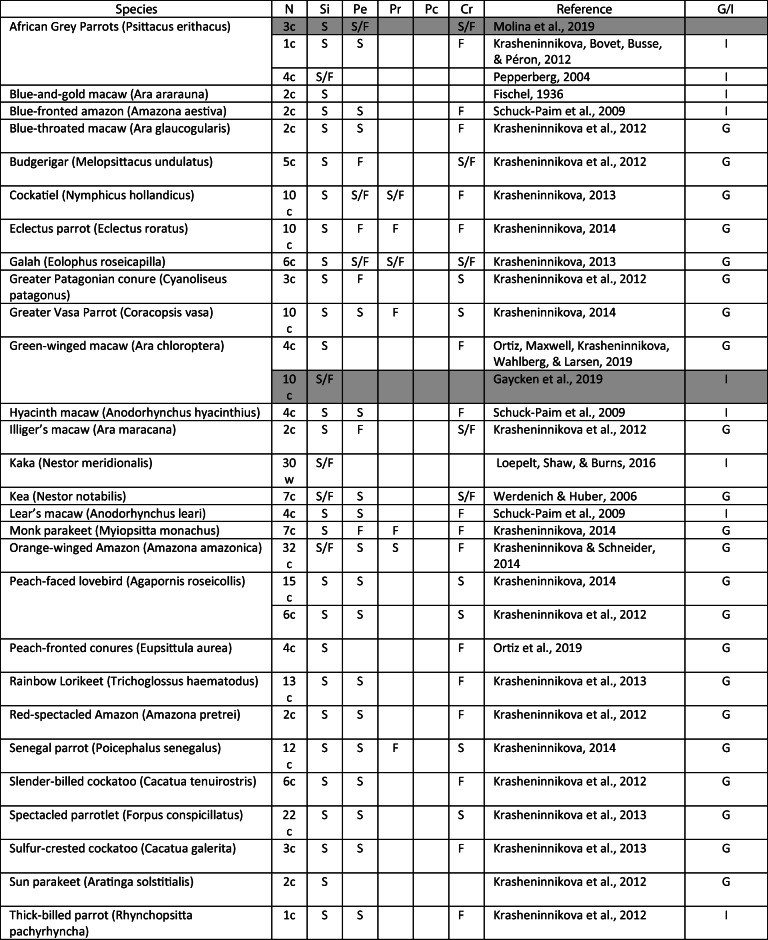
Twenty-eight parrot species have been tested in various vertical string-pulling patterns; here, we report only the performance in conditions also conducted in the present study

*Note. N* = number of individuals (c = captive, w = wild); Si = single string (baseline); Pe = perpendicular; Pr = proximity; Pc =perception; Cr = crossed; S = success; F = failed; G/I indicates a study’s success determined on group level or individual level; highlighted in grey are studies employing visual limitations

Goffin’s cockatoos (*Cacatua goffiniana*) are feeding generalists (Mioduszewska et al., 2019; O’Hara et al., 2019), originally endemic to the Tanimbar Islands in Indonesia. Studies under controlled laboratory conditions have highlighted this species’ proficiency in several technical tasks, requiring abilities that have been argued as prerequisites to succeed in string-pulling (see Auersperg, Teschke, & Tebbich, 2017; Jacobs & Osvath, 2015): They react flexibly and sensitively to small changes in their environment (Auersperg, Borasinski, Laumer, & Kacelnik, 2016; Auersperg, Kacelnik, & von Bayern, 2013; Auersperg, Laumer, & Bugnyar, 2013), they seem to attend to several functional aspects while solving physical tasks (Auersperg et al., 2013), including the relations between objects (Habl & Auersperg, 2017), and, most notably, they exhibit some degree of means–end apprehension during the manufacture and use of tools (Auersperg, Szabo, von Bayern, & Kacelnik, 2012; Auersperg et al., 2014; Auersperg, Köck, Pledermann, O'Hara, & Huber, 2017; Laumer, Bugnyar, Reber, & Auersperg, 2017). However, despite the extensive research in regard to their physical cognition, this species has not been previously tested on any of the classic variations of the string-pulling paradigm. Our aim was to close this gap by providing naïve Goffin’s cockatoos with a classical string-pulling setup with a single string, as well as versions using perpendicular, crossed, shortened, and coiled strings, in addition to adopting a slightly modified design to control for visual access.

Taking into consideration other parrot species’ past performances (above), we expect that Goffin’s cockatoos possess the basic sensorimotor skills required to spontaneously solve a single string condition without any visual restriction. To investigate whether the cockatoos could act in a goal-directed manner, we used two strings of the same length with only one string rewarded (perpendicular condition) to see whether they chose the baited string immediately and above chance expectation, or if they pulled randomly, either due to a lack of attention to the end of the strings or a desire to explore the strings. In order to see whether the subjects followed a spatial proximity rule, we used two rewarded strings, with one shorter than the other (proximity condition), expecting them to choose the shorter (and therefore closer) string over the longer string if they were using such a rule. Visual access to the string was prevented in this condition to keep the setup consistent between tests. This setup allowed for testing of the proximity rule, in contrast to using the more commonly slanted or crossed strings as these test setups would only allow for proximity rule testing in case the leading of the string could be perceived. Random choice in this condition would suggest a lack of sensitivity to spatial proximity, whereas choosing the longer string above chance could indicate that the action of string-pulling may in itself be intrinsically rewarding (e.g., exploration or play). To see if the cockatoos were making choices based on proprioceptive rather than visual feedback, the baited string was left long enough to be coiled onto the apparatus floor, and the unbaited string was both weighted and shorter (coiled condition). If this heavier string was repeatedly chosen, it would indicate that attention was focused on proprioceptive feedback, as the coiled string did not offer any perceptive weight resistance during the first pulling actions. It was expected that individuals would choose the rewarded coiled string over the unrewarded standard-length string with weight resistance if their choice was based on visual rather than on proprioceptive feedback. The chosen setup of the coiled condition shed light on the perceptive abilities of Goffin’s cockatoos when presented with both strings long and coiled but one of these having a broken connection, as neither of the strings would provide any perceptual weight resistance within the first pulling actions. Finally, in order to investigate representation, the subjects were presented with two crossed strings, with only one rewarded (crossed condition). Due to the board acting as a visual occluder, subjects could only inspect the crossed string setup while perching in front of the apparatus before the start of each trial, but not from above while performing the pulling behavior. Therefore, if the birds were able to maintain a mental representation of the crossing during their decision-making process, we would expect the rewarded string to be chosen immediately and more than expected by chance; on the contrary, choices based on visual proximity would result in the pulling of the nonrewarded string. Random choices could indicate that contradicting information (immediate visual feedback vs. previous experience) resulted in conflicting motivation, or the adoption of cognitively less demanding, yet beneficial, strategies, such as side biases. Finally, if the proximity, coiled, or crossed conditions were intrinsically rewarding, we would expect to find no preferential or above chance choices.

## Methods

### Subjects

This study was conducted at Goffin Lab Goldegg of the Messerli Research Institute in Austria, which houses a group of 16 hand-reared Goffin’s cockatoos (see Table 2) in a flock with an indoor and outdoor area (indoor: 45 m^2^, 3–6-m height; outdoor: 150 m^2^, 3–6-m height). The birds had ad libitum access to fresh water and food (variety of food pellets, fruits, vegetables, and mineral supplements). Nine individuals participated in the double string conditions and an additional three individuals participated in the single string test (*n* = 12).

**Table 2:** Detailed subject information: names, sex, and hatch date of the 12 participating cockatoos and information about participation in single and all conditions with two strings, as well as the number of trials required to reach a criterion of 10 consecutive correct choices during training

Name	Sex	Hatch date	Single string test	Training trials	Double string test
Figaro	Male	2007	Yes	110	Yes
Fini	Female	2007	Yes	310	Yes
Pipin	Male	2008	Yes	250	Yes
Heidi	Female	2010	Yes	250	Yes
Zozo	Male	2010	Yes	40	Yes
Kiwi	Male	2010	Yes	250	Yes
Konrad	Male	2010	Yes	–	No
Dolittle	Male	2011	Yes	310	Yes
Mayday	Female	2011	Yes	–	No
Jane	Female	2017	Yes	100	Yes
Titus	Male	2017	Yes	190	Yes
Irene	Female	2017	Yes	–	No

Cashew nuts were only available as a reward during test sessions, in which the cockatoos participated voluntarily. All subjects had CITES certificates and were registered at the district’s administrative animal welfare bureau (Bezirkshauptmannschaft St. Pölten, Schmiedgasse 4–6, A-3100, St. Pölten, Austria). These housing conditions were following the Austrian Federal Act on the Protection of Animals (Animal Protection Act—§24 Abs. 1 Z 1 and 2; §25 Abs. 3—TSchG, BGBl. I Nr. 118/2004 Art. 2). As our tests were appetitive, noninvasive, and based exclusively on behavioral observations, they were not classified as animal experiments under the Austrian Animal Experiments Act (§2. Federal Law Gazette No. 501/1989). Although research at Goffin Lab Goldegg has mainly focused on physical cognition, only three individuals had some prior experience in a double string-pulling task, all other subjects were string-pulling naïve. The subadults Jane, Titus, and Irene were introduced to a double string-pulling task at an early ontogenetic stage by providing them with vertical and horizontal strings (length = 25 cm). One string had an object connected to one end, whereas the other string did not. In the course of a 24-weeks-development test, the three birds managed to successfully pull in the baited string within three consecutive trials.

### Apparatus

**Fig. 1:**
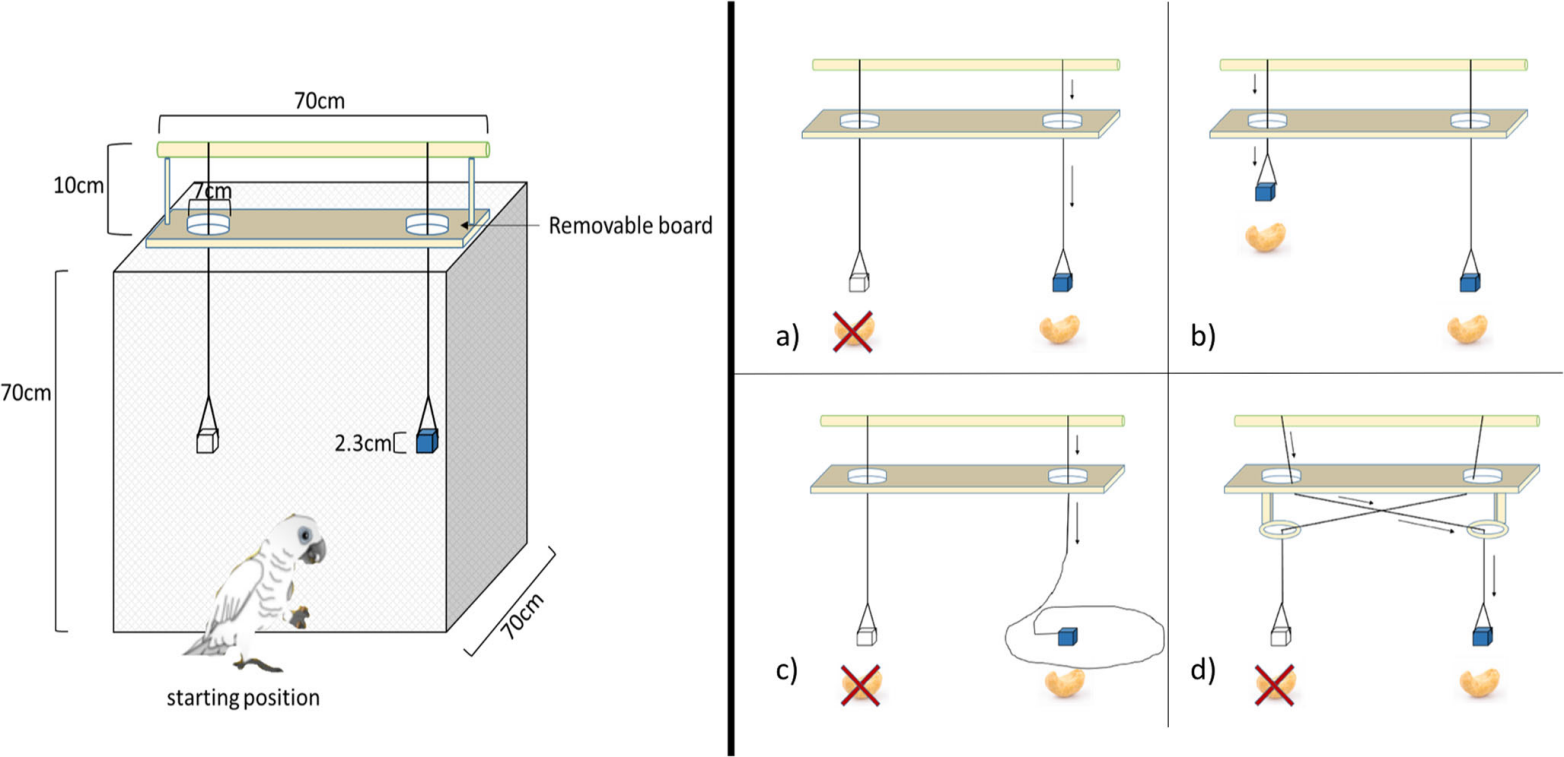
Illustration of the apparatus used with measurements (left). Arrangement of strings in different conditions (right) with the blue cube representing rewarded containers and the white cube containers without a reward. Arrows show the insertion direction of the string. **a** Perpendicular (baseline) condition: two strings both 70 cm in length. **b** Proximity condition: two strings, 70-cm and 35-cm long, both baited. **c** Coiled condition: two strings 70-cm and 100-cm long, the longer one baited and being coiled on the bottom of the box. **d** Crossed condition: two strings, both 85 cm in length, held in a crossed position by ring-shaped pieces of plastic; note that visual access to the crossing of the strings from above is prevented by the board

The apparatus consisted of a cubed wire cage that enabled subjects to pull the strings through holes in the top. The strings were inserted starting from the perch through a board with two holes (see Fig. 1) to ensure a restricted view of the strings by the birds. The subjects could view the inside of the apparatus from their starting position in front of it.

Customary jute strings with a diameter of 2 mm were used, and they varied in length depending on the chosen condition.

Cube-shaped containers with a side length of 2.3 cm and an open top, to enable a clear view of the contents, were attached to the ends of the strings. The container holding the reward was colored blue, while the empty container remained transparent. A shim was glued to the bottom of both containers to increase their weights to 10 g.

The subjects were trained to differentiate between the two cubes and to prefer the blue colored one over the transparent one, by offering each subject both cubes at the same time on a white table without any attached string; visual access to the inside of the cubes was prevented. The cockatoos had to choose the blue cube over the transparent cube five times in a row to ensure an association had been established.

### Procedure

The cockatoos were habituated to the apparatus in groups of five to eight birds by providing small pieces of pecan nuts and parrot baby mash. Later, they were individually encouraged with a food reward to sit on the perch on top of the box. Four different double-string conditions were used to test different cognitive abilities described in this section, plus a single-string condition to test for spontaneous string-pulling. The cockatoos were able to view the cubes from the perch in front of the apparatus as well as through the holes on top at all times. Nevertheless, their view of the strings was obstructed from the top of the apparatus in all double-string conditions. The view of the reward within the container, however, was not restricted.

Table 3 details the criteria for a trial being determined as successful in both the single-string and double-string setups; for all four double-string conditions, the same criteria had to be met.

**Table 3:** Detailed information on the condition, requirement, and criteria to be met determining a trial as successful for the single-string and all double-string conditions

Condition	Requirement	Criteria to be met
1 string	Spontaneous pulling	Spontaneously pull a string of ≥ 40 cm without prior training within 5 minutes
	Start of next trial	As soon as the setup has been prepared (pause between trials not necessarily required but permitted)
2 strings	End of trial	≤2 minutes from the start signal for individuals not beginning to solve the task (waiting)
		≤5 minutes from the start signal for individuals working to solve the task (working)
	Choice of string	Correct string must be chosen first (only a touch of the unrewarded string first without any pull with beak-foot-beak sequence permitted)
	Pulled string	Correct string must be pulled until the blue colored container is touched by the subject’s beak
	Reward taken	In cases where the reward was lost due to the pulling efforts, it was provided by the experimenter as soon as the blue colored container was touched by the subject’s beak (in the proximity condition, the one on the short string)
	Start of next trial	As soon as the setup had been prepared (pause between trials not necessarily required but permitted)

### Single string

The cockatoos’ sensorimotor abilities and spontaneity were tested by offering them one baited 70-cm long string in an apparatus without the board on top that would restrict their view into the box.

### Double-string test

#### Perpendicular condition (baseline condition)

Two strings of the same length of 70 cm were used, one of them baited and the other one not (see Fig. 1a). This condition was for testing goal-directedness and, if the birds continued to choose at random in the beginning, they were trained to succeed before starting the next condition as listed in Table 2, Training Trials column. This was done because the perpendicular condition served as baseline and a precondition to start the other three double string conditions (see below) in order to control for their basic understanding of a double-string task and therefore their goal-directedness in their further decisions and actions in the experiment.

#### Proximity condition

Two strings of different lengths, one 70 cm and the other 35 cm, were used, both of which were baited with pieces of cashew nuts (see Fig. 1b). The subjects’ susceptibility to proximity was tested in this condition.

#### Coiled condition

Two strings of different lengths were used, one which was 100 cm with 25 cm of its end coiled on the bottom of the box and baited, and one that was 70-cm long, which was not baited (see Fig. 1c). The purpose of this condition was to test for a potential reliance on proprioceptive or visual feedback (e.g., whether the cockatoos kept pulling the visibly baited coiled string without feeling the weight of the reward container for the first two to three pulling occurrences). The number of pulling actions depended on their body length.

#### Crossed condition

Two 85-cm long strings were led into a crossed position by a ring-shaped piece of plastic (see Fig. 1d). As the crossing of the strings was visually restricted by the top board, this condition tested the subjects’ potential to act on a previously acquired mental representation of the setup, as opposed to relying on immediate visual feedback.

### Training procedure

#### Single string

After successful habituation to the apparatus, the birds were tested for their spontaneity with the single-string condition. The cockatoos had to succeed in pulling the string in three sessions of 12 trials each before performing the two string training sessions for the baseline condition (perpendicular).

In case a bird did not succeed in pulling the 70-cm string, the string was shortened to 40 cm. After five consecutive successful trials, the string was set to 55 cm for another five consecutive successful trials before setting the string back to 70 cm for three sessions of 12 trials each.

#### Training procedure: perpendicular (baseline)

To ensure that individuals were sufficiently familiar with the affordances of the visual restriction, we presented the perpendicular condition, including the board installed on top of the apparatus, as part of the training. In case the birds did not successfully choose the baited string for 10 consecutive trials within 16 sessions, both strings were shortened to 40 cm, and the board on top of the apparatus, which was restricting the subjects’ view of the course of the strings, was removed. The birds performed 10 trials before the strings were expanded to 70 cm for another 10 trials, still without a board on top of the apparatus.

The next step was a session of 10 trials with 70-cm strings after the board was installed on top of the box, which allowed the subjects to see the containers at the end of the strings for another 10 trials. In case the subjects did not succeed after this session, the procedure was repeated.

Training for the perpendicular condition was complete after 10 consecutive successful trials with 70-cm strings and the board installed on top of the apparatus, restricting the subjects’ view from above and only allowing sight of the containers at the end of the strings (see Table 2 for the number of trials until this criterion was met).

### Testing

All tests were started at the front of the box with a clear view of the task arrangement for the subject. The experimenter started every trial verbally with a start signal, after which the birds voluntarily jumped or flew on the top of the apparatus.

The maximum duration of a trial was 2 minutes. In case a subject did not start the trial within this timeframe, it was considered unsuccessful due to time-out. If the bird started to work on the trial but did not succeed in pulling up the string with the reward successfully within 2 minutes, the duration was extended to 5 minutes before the trial was considered unsuccessful.

Nine cockatoos participated in 12 sessions, each consisting of 12 trials. Each condition involving two strings was provided three times per session. The order of the conditions within each session was randomized for every bird. The laterality of the rewarded strings was semirandomized per trial for every subject, to prevent the rewarded string being presented on the same side more than five times in a row within the same session.

Testing in the single string condition was limited to a maximum of 3 days per week between November 11, 2018, and December 1, 2018; the individuals received one session per testing day. Testing in the

double-string conditions was limited to a maximum of 4 days per week between September 29, 2019, to February 13, 2020. Most individuals performed one session per testing day. However, no more than two sessions per individual were given on the same testing day.

During testing, the experimenter wore mirrored sunglasses to prevent gaze following. All trials were recorded using a camcorder.

### Analysis

The analysis was performed using R Version 3.6.3 (R Core Team, 2018). We examined the performance of all cockatoos per condition using a binomial mixed model, including condition, side of rewarded string, trial, and session as main effects, and subject as a random factor with the lme4 package (Bates, Mächler, Bolker, & Walker, 2015). Model selection was performed by stepwise model reduction based on the Akaike’s information criterion (AIC). Post hoc analysis of differences between conditions was carried out with Tukey pairwise comparisons employing the package multcomp (Hothorn, Bretz, & Westfall, 2008). To correct for multiple comparisons, results were adjusted using a Bonferroni correction. Individual performance, as well as the side of choice within each condition, was assessed using one-sided binomial tests to evaluate whether the probability of the observed performances was greater or less than would be predicted by chance.

## Results

### Single-string condition

Nine out of 12 cockatoos started to pull a single string of 70 cm after some exploratory actions (nibbling the string or the perch); eight of them immediately performed the targeted action of pulling with the beak, holding the string with the foot, and repeating these behaviors (pull-hold-pull) without prior training. The other three individuals obtained the reward during their first attempt pulling a string of 40 cm instead of 70 cm. One individual, Pipin, in his first attempt with a 40-cm string, once took some steps sideways, holding the string in his beak until the reward could be obtained, similar to the pulling technique of some cockatiels and kea (Krasheninnikova, 2013; Werdenich & Huber, 2006). However, as of the second trial, he switched to the pull-hold-pull technique, which continued for the remainder of the trials. Seven of the nine individuals who initially pulled the 70-cm string successfully obtained the reward in their very first attempt. Both Dolittle and Titus explored the string and tried to pull it with their beak, but did not hold it with their foot to obtain the desired reward, while Figaro did not even approach the string at all in his initial 70-cm string trial. Using the 40-cm string setup, all three immediately performed the pull-hold-pull sequence successfully. The subadults Irene, Jane, and Titus, as well as one adult, Zozo, pulled the string while sitting on the perch on top of the box in their initial attempts, while all other individuals preferred to remain sitting on the wire grid. However, during the course of the experiment, all of the Goffin’s cockatoos ended up pulling the string from the wire grid instead of the perch, though their technique remained the same regardless of their position.

### Double-string conditions

Three of the 12 birds that participated in the single string condition dropped out of the subsequent double string tests, leaving nine subjects for the remaining conditions.

The binomial generalized linear mixed model revealed no influence of trial (GLMM). *χ*^2^(11) = 14.09, *p* = 0.23, or session (GLMM), *χ*^2^(11) = 6.04, *p* = 0.87, on the choice of strings. However, we did find a significant effect of condition (GLMM), *χ*^2^(3) = 481.78, *p* < .001, on successful performance. Post hoc analysis revealed significant differences between all levels of condition (see Table 4).

**Table 4:** Results of the pairwise Tukey post hoc test comparing all levels of condition; error-probability has been adjusted for multiple comparisons employing the Bonferroni-correction method

Condition	β	*SE*	*z* value	*p*	Sig.
Crossed vs. coiled	−2.9423	0.2121	−13.872	<.001	***
Perpendicular vs. coiled	1.2005	0.2215	5.420	<.001	***
Proximity vs. coiled	−0.7348	0.1695	−4.335	<.001	***
Perpendicular vs. crossed	4.1427	0.2510	16.507	<.001	***
Proximity vs. crossed	2.2074	0.2042	10.808	<.001	***
Proximity vs. perpendicular	−1.9353	0.2155	−8.979	<.001	***
Signif. code	.001 ***				

**Fig. 2:**
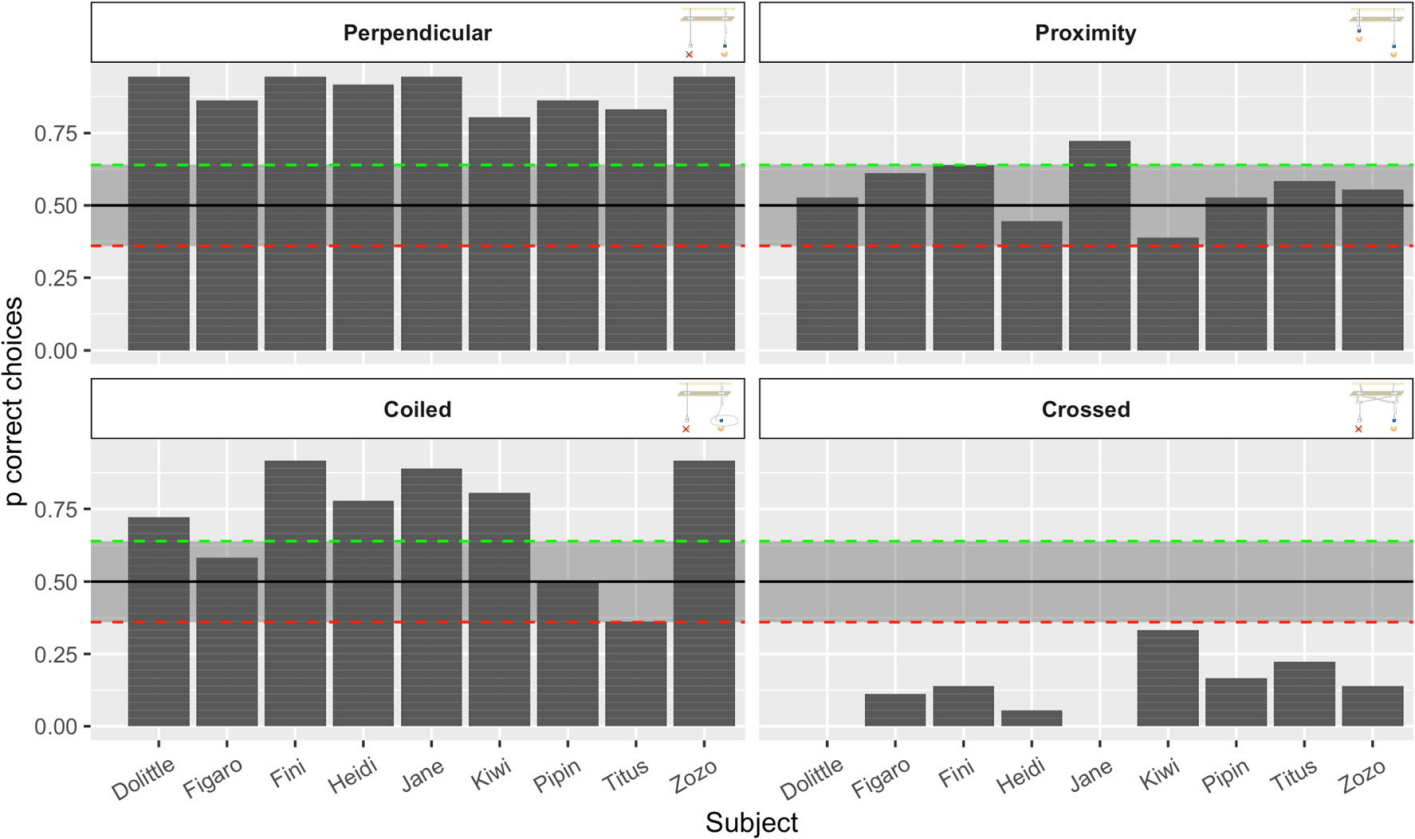
The observed proportion of correct choices per individual shown by condition; solid black line and grey bounding box indicate performance at chance level; dashed lines demark borders of significantly less (red; probability of success = 0.36, *p* = .066) and more (green; probability of success = 0.6, *p* = .066) correct choices than predicted by chance. (Color figure online)

Analysis of the number of correct choices using the binomial test on an individual level (see Fig. 2) revealed that each subject chose the baited string significantly above chance in the perpendicular condition, whereas every individual chose the unbaited string in the crossed condition significantly above chance. Two individuals, Jane and Dolittle, refused to work in the crossed condition and did not attempt to pull any of the two strings throughout all sessions. Only one individual, Jane, chose the reward on the shorter string significantly above chance in the proximity condition, with all other subjects showing no preference for a particular string. Six out of nine subjects chose the correct string in the coiled condition significantly more often than predicted by chance, while two individuals performed at chance levels. One individual, Titus, exhibited a tendency (*p* = .067) to pull on the short, but unrewarded, string more often.

**Fig. 3:**
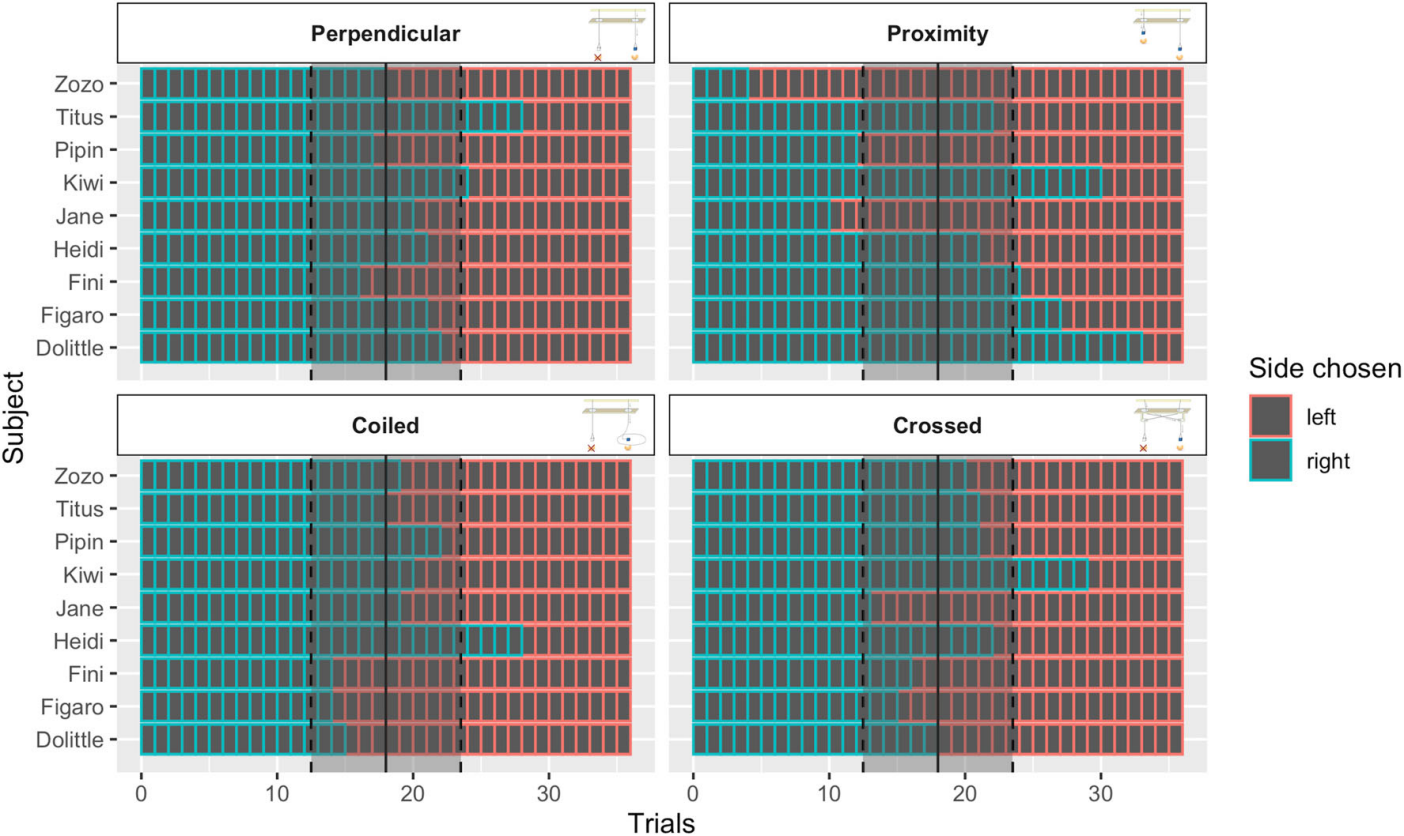
The observed proportion of sides chosen by each individual shown within each condition. Solid black lines and grey bounding boxes indicate a nonsignificant choice of one side over another; dashed lines demarcate strings significantly chosen more often on the right side (red) or on the left side (turquoise). (Color figure online)

Furthermore, our model highlighted a significant effect of reward position on the number of correct choices (GLMM), *χ*^2^(1) = 16.99, *p* < .001. We performed individual binomial testing of the side selected irrespective of reward position (see Fig. 3). Only one individual, Titus, exhibited significantly more pulling behavior on the right side in the perpendicular condition. Similarly, only one bird, Kiwi, was observed to pull more often on a particular side in the crossed condition (right), and only one subject, Heidi, chose the right side more often in the coiled condition. However, seven out of nine individuals pulled the string significantly more often on one particular side in the proximity condition, and only two individuals showed no side preference. Three individuals (Zozo, Pipin, and Jane) exhibited a left-side bias, and four individuals (Kiwi, Fini, Figaro, and Dolittle) chose the right string significantly above chance levels.

## Discussion

We aimed to test naïve captive Goffin’s cockatoos on a specific set of conditions using the string-pulling paradigm (see below). Considering that seven out of 12 subjects solved the single-string condition on their first trial, we may conclude that they are capable of immediately employing the appropriate set of requisite motor actions (Werdenich & Huber, 2006). Pulling in out of reach items might be part of their foraging repertoire in their natural habitat.

**Fig. 4:**
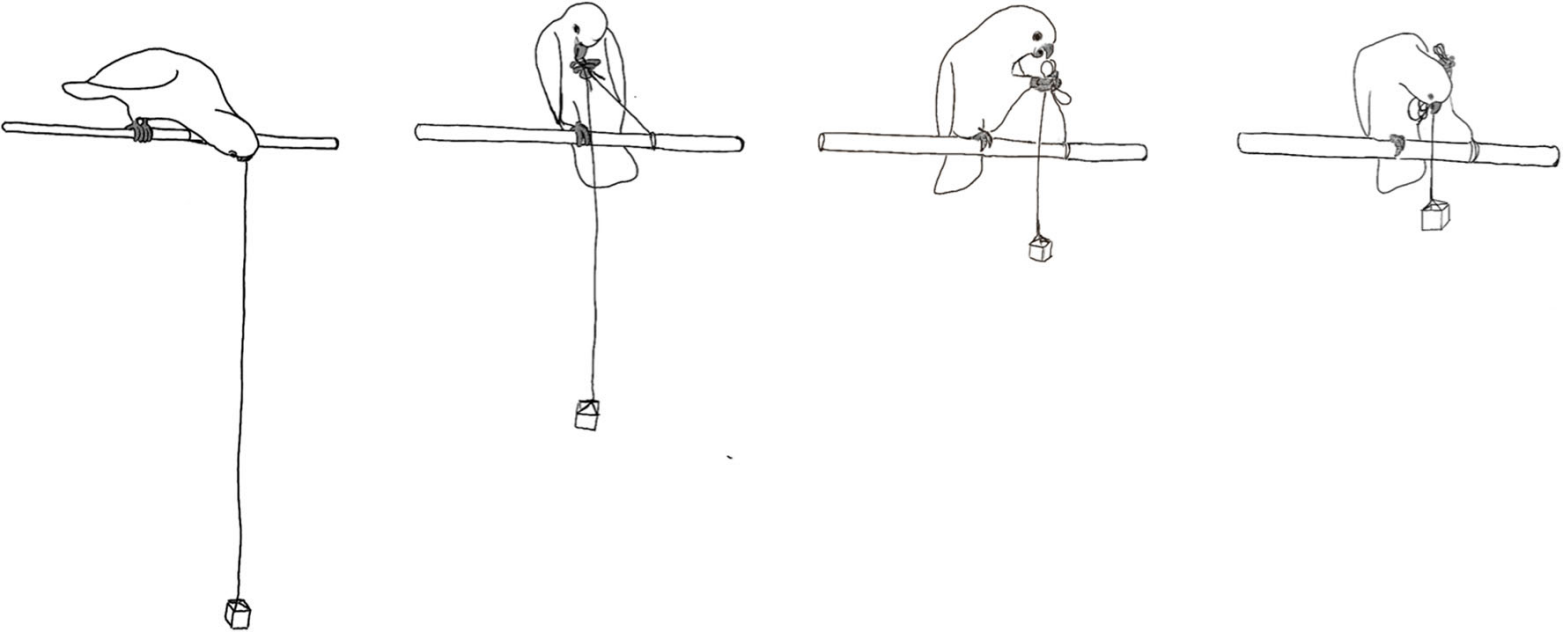
String-pulling sequence as performed by subject Irene (Illustration by Mark O’Hara)

The pulling technique used by the cockatoos differed substantially from previous observations in some other parrot species, such as cockatiels (Krasheninnikova, 2013) and grey parrots (Pepperberg, 2004). While the former succeeded by sliding, flipping, looping, side walking, or turning (Krasheninnikova, 2013), the Goffin’s cockatoos’ technique seemed more similar to that used by galahs (Krasheninnikova, 2013) and keas (Werdenich & Huber, 2006)—namely, pulling the string in an upright position. However, the galahs pulled the string with their feet (Krasheninnikova, 2013), whereas the Goffin’s cockatoos used their beaks for the pull-up action (see Fig. 4). Moreover, the kea performed the step-on method to hold the string in position during repetitive pulling bouts, while the Goffin’s cockatoos alternated between pulling with their beak and holding the string up with one foot to prevent it from falling back down. The Goffin’s cockatoos have highly developed beak–foot coordination up to the point of being able to open a bolt with their feet (Auersperg et al., 2013). Although two kea used the technique of grasping and pulling the string with the foot (Werdenich & Huber, 2006), they did not grab the string with the beak and hold it in place with their foot, as observed with the Goffin’s cockatoos. However, the upright pull, as described as being used by some kea (Werdenich & Huber, 2006), was also performed by Goffin’s cockatoos.

Using the double-string conditions, we hoped to more closely examine the four criteria of means–end understanding as formulated by Jacobs and Osvath (2015): (1) goal-directedness, (2) no proximity error, (3) flexible solutions, and (4) no dependence on immediate feedback.

Unsurprisingly, all cockatoos exhibited (1) goal-directedness in the perpendicular condition after training. The criterion (2) no proximity error was met by all individuals, except for the subadult Jane, who pulled the shorter string more often than expected by chance. Hence, it is unlikely that subjects paid attention to the distance of the reward. Either the birds did not differentiate between the efforts of pulling a longer or shorter string or, not mutually exclusively, the effort to pull a long string was not sufficiently discouraging. However, the length of the long string was double the length of the short string, and thus comparable to a setup in Western scrub-jays (Hofmann et al., 2016). The Western scrub-jays did not show a preference for the shorter and therefore more efficient of two strings in their initial choices, but did so in their final choice in two of the three tasks, because they showed a high proportion of switches to the short string when their initial choice was the long string. An alternative explanation could be that the string-pulling behavior may be rewarding in itself (see Mason, Harlow, & Reuping, 1959; Schuck-Paim et al., 2009).

While criterion (3), flexible solutions, was not a focus of this present study, we examined criterion (4), no dependence on immediate feedback, with the coiled condition. Six out of nine individuals performed above chance with only one subadult male (Titus) showing a tendency to perform below chance expectation (*p* = .067). While the latter bird’s tendency to choose the shorter string may suggest that he made his decision based on the proximity of the string’s end, the success of the majority of birds in the coiled condition allows us to exclude that there is a predominant reliance on proprioceptive feedback for solving this task.

The final condition involved crossed strings that were out of immediate sight during pulling. As all individuals performed significantly below chance expectation, we presume that Goffin’s cockatoos most likely focused on the immediate visual feedback provided through the holes of the board on top of the box. They did not seem to memorize and retrieve the visual information they received while sitting in front of the box once they were lifted up to the top. However, it is noteworthy that one individual (Pipin) spontaneously solved the crossed condition correctly in his very first trial and throughout the first session, though his efficiency decreased substantially during the experiment. This supports previous findings showing that Goffin’s cockatoos based their choice between two tool locations on task information gathered prospectively (briefly seeing a task before the two tools), but did not manage to apply similar task information gathered retrospectively (briefly seeing the tools before the task; Beinhauer, Bugnyar, & Auersperg, 2019). Subjects might have perceived the affordances of the crossed strings, but did not consider it a task before they had access to the strings, and thus were not sufficiently motivated to attend to the setup. Repeating this condition with additional naïve individuals without visual restriction and evaluating early trial performance might thus provide further insights into the formation of mental representations of the setup.

Difficulties in the crossed-string condition are consistent with the results of most similar studies with other parrot species (see Jacobs & Osvath, 2015, and Table 1 for a review). Nevertheless, again, we must highlight that, unlike other previous successful parrot studies, in our study the crossing of strings was visually blocked by the top board of the apparatus during the string-pulling action. Thus, the crossing could have only been perceived before accessing the perch, and any correct choice would have had to be made based on a mental representation of the task configuration. As none of the individuals changed their strategy after unsuccessful trials, reliance on visual proximity dominated over any potential short-term learning effect. This may be explained by an overtraining or carryover effect (Díaz-Uriarte, 2002; Mandler & Goldberg, 1975) from the training phase. The individuals likely learned to predominantly focus on the configuration from the top, rather than paying attention to the arrangement form the starting position. As we found no learning effect for the cockatoos, neither within one session nor from one session to another, we think it likely that the birds reexamine the task setup prior to every trial and did not conclude a solution from past trials or sessions. Future replication of this study without prior training may be informative if reliance on direct visualization was acquired during the training stage.

Despite trying to control for the development of a side bias by pseudorandomizing the position of the reward, several individuals exhibited side preferences. Such a strategy can be beneficial as they are rewarded in 50% of the cases (Gagne, Levesque, Nutile, & Locurto, 2012; Holekamp, Swanson, & Van Meter, 2013; Jacobs & Osvath, 2015). It has been shown that individual birds developed side biases in different conditions, indicating cognitive difficulties with specific task properties (O’Hara, Auersperg, Bugnyar, & Huber, 2015). Side biases in the proximity condition may also suggest that the effort of pulling a longer string was not sufficiently cumbersome.

While the abilities required for string-pulling, such as Gestalt perception, causal reasoning and means–end understanding, have been argued to play a role in extractive foraging, nest building, and tool use (reviewed in Auersperg et al., 2017), the ecological relevance for a wild animal to perform a string-pulling task is rarely discussed; to our knowledge, a direct example where this skill would be applied in nature has not been described so far for parrots. Halsey, Bezerra, and Souto (2006) discussed observations of wild common marmosets (*Callithrix jacchus*) attempting to access out-of-reach fruit, and place string-pulling capacities within a foraging context for primates. As for parrots, Magat and Brown (2009) showed that species that used their feet for manipulation during foraging were more likely to succeed in a string-pulling task. Similarly, Krasheninnikova (2013) discussed intricate foot–beak coordination as a potential adaptation to the foraging ecology. Exploratory play during ontogeny was considered as another factor that contributed to developing capacities involved in string-pulling on a proximate level. We agree that most likely play (on a proximate) and foraging ecology (on the ultimate level) have allowed parrots to develop the skills necessary to solve the string-pulling paradigm. Here, we also provide a direct example from the wild in which string-pulling skills may be adaptive.

In their natural habitat, the small Tanimbar archipelago in the Moluccan region of Indonesia, the Goffin’s cockatoos feed on wild maracuja (*Passiflora foetida*; Mioduszewska et al., 2019; O’Hara et al., 2019). These flowering plants produce small individual fruit pods growing on long vines and represent a highly valuable foraging source to the wild cockatoos. The birds mostly prefer the ripe yellow fruit that may grow alongside unripe green fruit on vines, which can grow up to five meters long (Witt & Luke, 2017). Multiple vines often occur together in shrubs and form entangled accumulations. To reach the prized fruit, the bids have to either descend on these vines or pull them up. This observation provides a direct example for ecological relevance of basic sensorimotor skills. As two of the subjects in this study were tested as juveniles and showed competence for string-pulling, we expect this skill to develop during early stages of their life. This is in line with findings in ravens that plateaued at Stage 5 in object permanence trials within the first 6 month during ontogeny (Bugnyar, Stöwe, & Heinrich, 2007).

Our results indicate that Goffin’s cockatoos possess the basic motor-sensory means required to succeed in the string-pulling task (Jacobs & Osvath, 2015). While subjects most likely relied on immediate visual feedback in the current study, these birds may still possess the relevant capacities to understand means–end relations and to base their choices on mental representations. However, such cognitively demanding mechanisms might be easily overruled by simpler ones (O’Hara et al., 2015), especially provided prior reinforcement. Therefore, further studies without prior training and additional conditions including visual access during choice in the crossed condition might help to resolve this ambiguity. Furthermore, a comparison of string-pulling abilities of captive and wild Goffin’s cockatoos would be beneficial to better understand whether cognitive abilities underlying string-pulling tasks develop with natural experience, and to examine the role of string-pulling in a socioecological context.
